# Olfactory Perception in Relation to the Physicochemical Odor Space

**DOI:** 10.3390/brainsci11050563

**Published:** 2021-04-28

**Authors:** Antonie Louise Bierling, Ilona Croy, Thomas Hummel, Gianaurelio Cuniberti, Alexander Croy

**Affiliations:** 1 Institute for Materials Science, Technische Universität Dresden, 01062 Dresden, Germany; gianaurelio.cuniberti@tu-dresden.de (G.C.); alexander.croy@tu-dresden.de (A.C.); 2 Department of Psychotherapy and Psychosomatics, Technische Universität, 01062 Dresden, Germany; ilona.croy@uni-jena.de; 3 Department of Biological and Clinical Psychology, Friedrich-Schiller-University of Jena, 07743 Jena, Germany; 4 Smell and Taste Clinic, Department of Otorhinolaryngology, Technische Universität, 01062 Dresden, Germany; thomas.hummel@tu-dresden.de

**Keywords:** physicochemical odor space, olfactory perception, molecule structure

## Abstract

A growing body of research aims at solving what is often referred to as the *stimulus-percept problem* in olfactory perception. Although computational efforts have made it possible to predict perceptual impressions from the physicochemical space of odors, studies with large psychophysical datasets from non-experts remain scarce. Following previous approaches, we developed a physicochemical odor space using 4094 molecular descriptors of 1389 odor molecules. For 20 of these odors, we examined associations with perceived pleasantness, intensity, odor quality and detection threshold, obtained from a dataset of 2000 naïve participants. Our results show significant differences in perceptual ratings, and we were able to replicate previous findings on the association between perceptual ratings and the first dimensions of the physicochemical odor space. However, the present analyses also revealed striking interindividual variations in perceived pleasantness and intensity. Additionally, interactions between pleasantness, intensity, and olfactory and trigeminal qualitative dimensions were found. To conclude, our results support previous findings on the relation between structure and perception on the group level in our sample of non-expert raters. In the challenging task to relate olfactory stimulus and percept, the physicochemical odor space can serve as a reliable and helpful tool to structure the high-dimensional space of olfactory stimuli. Nevertheless, human olfactory perception in the individual is not an analytic process of molecule detection alone, but is part of a holistic integration of multisensory inputs, context and experience.

## 1. Introduction

Although the sense of smell is the evolutionarily oldest sensory system, many basic rules governing olfaction remain obscure to this day. As a chemical sense, olfaction relies on the sensory detection and perceptual interpretation of odorous molecules in the environment. A lot of research has tried to solve the intriguing question of the so-called “*stimulus-percept-problem*”: How does the molecular structure of an odor map onto its olfactory perception (for an excellent overview see [1])? Despite a steadily growing field of research on this topic, the underlying mechanisms of *whether* and *how* an odor is sensed and perceived based on its structure are still incompletely understood, in contrast, for example, to the visual or auditory domain. 

There are some well-known relations between structure and odor perception. In order to be perceived as odorous at all, a molecule must be volatile enough to evaporate and have specific solubility characteristics to pass through the (hydrophilic) nasal mucosa and bind to the (hydrophobic) olfactory receptors in the olfactory epithelium [2]. In addition, some functional groups have been associated with characteristics of odor quality. For example, many esters are known for a sweet or fruity odor and specific aldehydes are associated with the scent of grass or leaves [1]. The physical and chemical properties of odors presumably also influence the perceived intensity and concentration threshold at which an individual can detect them. Intensity is positively associated with vapor pressure, i.e., how many molecules are released into the air to reach olfactory receptors, and negatively relates to water solubility (hydrophilicity) [3]. Interestingly, the ability to judge odor intensity remains intact in humans with brain lesions, who are incapable of characterizing odorant qualities [4,5]. This suggests that intensity encoding may function independently of odor discrimination. In this context, it must also be taken into account that the perception of intensity, especially at high concentrations of odors, is influenced by both olfactory and trigeminal processing [2,6]. In addition, odor molecules with a high molecular weight were found to have higher rates of specific anosmia, not being able to smell a specific odor, than lighter molecules [6].

Probably the most extensively investigated perceptual dimension of olfaction is its hedonic valence, or *pleasantness*. Pleasantness has a special role in olfactory perception. Unlike vision, where this dimension plays a rather subordinate role, naïve subjects tend to respond to hedonic properties of odors before thinking about their quality or intensity [7]. Pleasantness was repeatedly found to be positively associated with molecular weight, size or complexity [3,8,9,10] and was highlighted as one of the most important dimensions of odor description [8,11,12,13].

However, direct relationships between specific physical or chemical characteristics with odor perception are rare, and structurally similar odor molecules in some cases lead to very different olfactory perceptions [1]. In addition, the understanding is complicated by many other processes in the pathway of olfaction, for example, influences due to chemical reactions during transport through the nasal mucosa or the interaction of odors in odor mixtures [1,2,14,15]. Thus, olfactory perception remains a “black box” in many respects. Ways to bridge this problem emerged with the advance of complex computational algorithms and modeling approaches. 

As a first step, many studies have attempted to characterize a “physicochemical odor space” [2,3,8,16]. For this purpose, a large number (typically thousands or tens of thousands) of chemical and physical molecular properties, so-called descriptors, are calculated using special software or online databases. To deal with the resulting high-dimensional property space, the most important dimensions are usually obtained using statistical decomposition methods such as principal component analysis (e.g., [8,17]). The resulting physicochemical odor space can then be used to study odor similarity [17,18] and qualitative or hedonic properties of odors [3,8,16]. For example, Khan et al. [8] were able to put novel molecules into the correct ranking of pleasantness according to their variance in the first component of the physicochemical odor space. This way, odorant pleasantness could be predicted with r~0.50 across three cultures [8]. Using sophisticated prediction algorithms, such machine or deep learning approaches, increasingly high prediction accuracies can be achieved without having to capture the complexity of all underlying interactions involved [19,20]. Keller et al. [16] even launched a crowd-sourced competition to obtain high prediction accuracies for their dataset with 49 study participants who rated perceptual dimensions of 476 odor molecules. 

One striking caveat of most of these experiments is, however, that the perceptual dimensions are obtained using ratings by olfaction experts, such as perfumers, wine tasters, etc. (e.g., see [7,21,22,23,24]). This procedure has benefits in a presumed higher objectivity and less interindividual variation in odor evaluation of the raters. Especially, when aiming to unveil the rules of odor *sensation* at the molecular level, small deviations in the ratings of the same odor are undoubtedly favorable. 

Many studies, on the other hand, are not aimed at a detailed understanding of processes on an atomic level, but focus on *perceptual* aspects of olfaction, i.e., finding reliable predictions of whether an odor is pleasant, familiar, intense, etc. In this context, one must ask to what extent expert ratings can be generalized to the population as a whole. Measures of olfactory perception, such as hedonic valence, vary between individuals—amongst others—due to influences of context and experience [25,26,27,28,29,30]. Consequently, they cannot be completely determined by structural composition alone. The question therefore arises as to how valid expert evaluations are when examining perception. As an analogy in the visual domain, one could compare this to asking art experts to judge the beauty of visual impressions. Although this might lead to more similar ratings than in a normal population sample, it can be asked if these are representative results. To date, there is a lack of studies that systematically address the relation between physicochemical structure and olfactory perception of “naïve” subjects. One of the few existing studies is the previously mentioned study by Keller et al. [3,16], who investigated an impressively wide range of chemically different odor molecules, but on only 56 individuals.

The purpose of this study is to investigate the extent to which previous findings about the relationship between odor structure and perception hold true in a sample of non-expert raters. To this end, we study the role of physicochemical properties for the *detection threshold* as well as for perceived *pleasantness*, *intensity* and *quality* of odors in a large naïve sample. We further examine the distribution and variance of the perceptual ratings to identify evidence of interindividual differences in olfactory perception. For that purpose, we reanalyze a dataset of 2000 subjects that rated 20 odors in a study by Croy et al. [6] and critically examine how perceptual ratings interact and vary between individuals. The findings intend to broaden the understanding of olfactory perception in relation to the physicochemical odor space and point out perspectives and challenges in the field.

## 2. Materials and Methods

**Figure 1:**
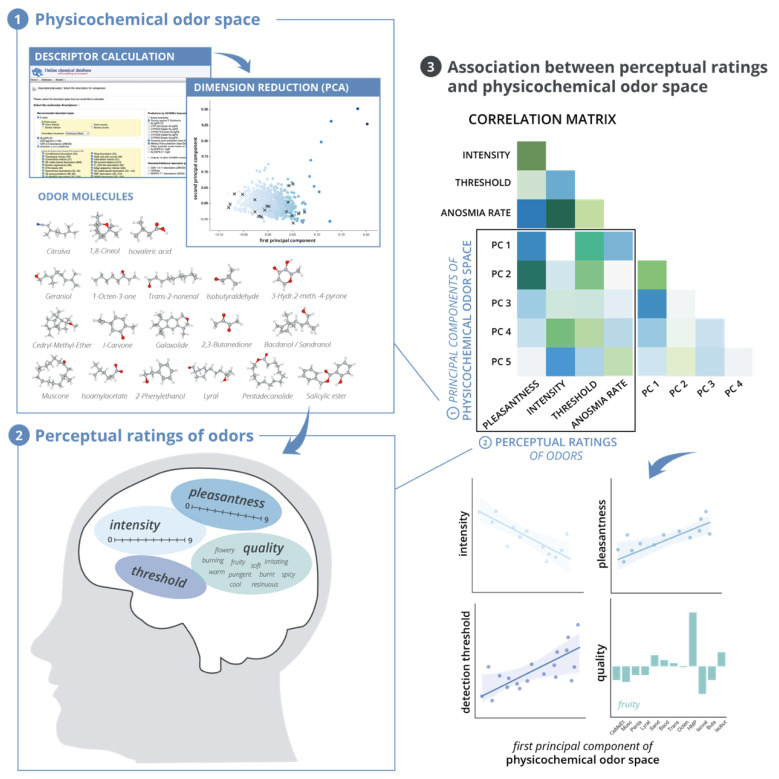
Overview of Methodology and Analyses. (**1**) Development of a physicochemical odor space. For 1389 odor molecules, including 20 molecules for which we obtained perceptual ratings (see 3D molecule structure images and (**2**)), molecular descriptors were calculated using the Online chemical database (https://ochem.eu/, accessed on 12 February 2021). After preprocessing of the dataset, dimension reduction was performed by the means of principal component analysis. The resulting components were used for further analyses (see (**3**)). (**2**) Perceptual ratings of odors. For 20 of the 1389 odor molecules, perceptual ratings were obtained from a dataset with *n* = 2000 subjects that were tested with one or more odors in groups of *n*~200 (Croy et al. [6]. The detection threshold and ratings of intensity, pleasantness and qualitative dimension were investigated for differences between the odors. (**3**) Association between perception and odor space. Finally, the values of the first principal components of the odor space for each odor were correlated with their corresponding perceptual dimensions of pleasantness, intensity and detection threshold. For the qualitative ratings, individual plots were generated showing the frequency of naming a specific qualitative descriptor for each odor molecule. Note: Plots show schematic visualizations. Three-dimensional molecules were drawn using VESTA software [33].

In order to investigate the relationship between odor properties and their corresponding perceptual impression in a large non-expert sample, we performed three main steps. At first, we followed previous approaches and developed our own physicochemical odor space as introduced in Khan et al. [8] (see Section 2.1). As a second step, we retrieved perceptual ratings [6] and investigated the distribution and differences in the ratings for 20 different odors (see Section 2.2 and Section 2.3). Thirdly, we correlated the results from the odor space with the perceptual ratings from our dataset. The procedure is visualized in a schematic overview in Figure 1.

### 2.1. Development of a Physicochemical Odor Space

#### 2.1.1. Descriptor Calculation and Preprocessing

For the development of a physicochemical property space of odors, we calculated physical and chemical descriptors for 1389 odors typically used in experiments and industry from a list provided by Khan et al. [8] For those odors, we first identified the corresponding molecule from its registry number or name using *webchem* package [31] in RStudio© (version 1.2.5033, R version 3.6.2). Afterwards, we used the *Online chemical database* (OCHEM, https://ochem.eu/, accessed on 8 December 2020; [32]) to calculate a total of 21,609 physical and chemical descriptors. For preprocessing, we removed all descriptors containing infinite or missing values for one or more of the molecules as well as descriptors with zero values for more than 80% of the molecules. Furthermore, to improve the quality of the principal component analysis, we dropped all descriptors without noteworthy correlations (no correlation of r>|0.3| with any other descriptor). This resulted in the final odor space with 4094 descriptors for each of the 1389 molecules. A more detailed report on the descriptor calculation can be found in Appendix A. All corresponding R and Python code and datasets for the analyses in this publication are available via https://osf.io/e67dn/ (accessed on 12 February 2021). 

#### 2.1.2. Dimension Reduction

To eliminate redundancies in the variables and reduce the dimensionality of the odor space, principal component analysis (PCA) was performed. PCA is a common method to reduce the dimensionality of large and complex datasets with high redundancies between variables. To achieve this, all data points are projected to new coordinates in a way that each dimension (=principal component) successively explains the largest amount of variance in the data and all resulting dimensions are orthogonal (i.e., uncorrelated) to each other. Usually, PCA requires more observations than variables in the dataset to yield robust estimates, which is not the case here since we have 4094 variables but only 1389 observations. A typical approach in cheminformatics, where this is a common problem, is the usage of the non-linear iterative partial least squares (NIPALS) algorithm [34]. The NIPALS method is based on finding linear combinations for each factor in an iterative way, starting with a randomly chosen starting vector. The procedure generates more precise results than the normally used singular value decomposition, but may also be slower if a large amount of components is calculated [34]. We performed PCA using the NIPALS algorithm on the 4094 molecular descriptors using *statsmodels* package as implemented in Python 3.7 [35]. The *statsmodels PCA* function first normalizes the data and then performs PCA on the desired number of components, ranging from one component to the number of variables in the dataset. To establish a reasonable calculation time, we chose to calculate 100 principal components. As a result, a matrix of factor scores was generated that reflects the position of each odor molecule in the odor space, and the corresponding factor loadings refer to the importance of each original descriptor for the principal components. In a last step, the factor scores for each odor molecule were stored in a data frame to be used for further analyses of associations between odor space and perceptual ratings (see Section 2.2).

### 2.2. Materials and Measures

For the analysis of perceptual ratings of odors, we used a dataset with 1600 participants from Croy et al. [6] that had originally been collected in three sub-experiments on prevalence and effects of olfactory training in specific anosmia, as well as yet unpublished data from another 607 participants of the same cohort. In the original study, each participant had been tested for odor detection threshold for one to seven out of twenty odorants (see Table 1 and Table 2) in nine dilution steps from 0 = 1:10^0^ to 9 = 1:10^9^. The (undiluted) odorants were diluted in 1,2-propanediol (CAS number 57-55-6). A total of 4 mL of each odor was presented in a 50 mL glass bottle with a diameter of the opening of 6 cm. Based on the results for the individual detection thresholds, the rate of specific anosmia was calculated, i.e., the percentage of participants not being able to smell the odor as operationalized by the deviation from the mean detection threshold. CAS number, trivial name, estimated vapor pressure and an abbreviate code for better readability in successive graphs for each molecule are provided in Table 1. For thirteen of the odors, participants had been asked to rate their individual perceptions of the highest odor concentration with respect to intensity, pleasantness and for twelve odors for a qualitative impression. Pleasantness and intensity had been rated using a scale from 0 through 9 (intensity: 0 = not perceived, 9 = extremely intense; pleasantness: 0 = extremely unpleasant, 9 = extremely pleasant). Qualitative ratings had been collected by asking the participants to choose two out of twelve verbal descriptors that, in their opinion, described the odorant best. For further details on the procedures, see Croy et al. [6].

### 2.3. Statistical Methods

All analyses were carried out in Jupyter notebook (see https://osf.io/e67dn/, accessed on 12 February 2021) using different packages in Python 3.7, including *Pandas*, *NumPy*, *Matplotlib*, *SciPy*, *statsmodel* and *Pingouin*. In order to detect significant differences between perceptual ratings for different odors, robust Welch one-way analyses of variance (ANOVAs) were performed for the factors pleasantness, intensity and detection threshold, and Games–Howell post hoc comparisons were calculated. The ANOVAs and post hoc tests were conducted using the *Pingouin* package (version 0.3.9) as implemented in Python 3.7 [36]. For the investigation of interindividual differences in the distributions of pleasantness and intensity ratings, the mean, median and standard deviations were calculated and the results visualized in histograms and boxplots. In addition, Pearson correlation coefficients were calculated for associations between the mean and standard deviations for the perceptual ratings, and we checked if the vapor pressure of the odors is correlated with the perceptual ratings using Spearman correlation coefficients. For the qualitative ratings, the percentage of naming the different verbal descriptors was calculated and visualized. Associations of the qualitative descriptions with mean intensity and pleasantness ratings were analyzed in an explorative fashion. Finally, Pearson correlation coefficients were calculated to investigate associations between the first five principal components of the odor space (see Section 2.1) with the perceptual ratings for the 20 odor molecules investigated. For those correlations, the rate of specific anosmia was added to compare the results to the previous findings in Croy et al. [6]. The graphs and visualizations of the results were built using the packages *matplotlib* and *seaborn* (Python 3.7.) in Jupyter notebook and were further processed in Adobe Illustrator 2021.

## 3. Results

### 3.1. Physicochemical Odor Space

#### 3.1.1. Factor Scores and Loadings

**Figure 2:**
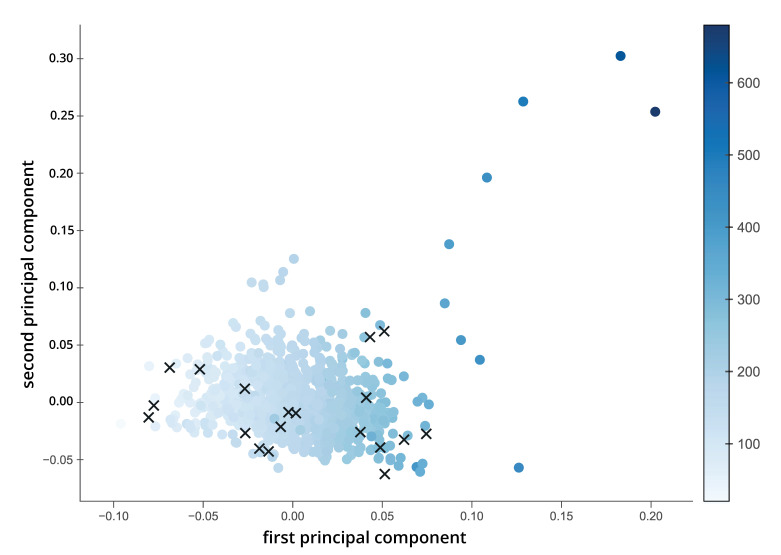
Physicochemical Odor Space. The graph shows the values for the first two principal components as obtained from dimension reduction of the physicochemical molecule properties for 1389 odor molecules. The 20 odor molecules used in the further analyses are highlighted as “x”. The shade of blue indicates the molecular weight of the molecule (g/mol).

For dimension reduction, we calculated 100 principal components from the 4116 original molecular descriptors (see Figure 2). Although a systematic analysis of the factor loadings is challenging due to the large number of descriptors, some associations and trends were explored, especially to check if previous results can be replicated. Similar to previous studies (e.g., see [2,3,8]), the first component showed a clear association with *molecular weight* (as shown by the shade of blue in Figure 2) and *complexity* (high loadings of *graph vertex complexity index* and *graph distance complexity index*; both descriptors from alvaDesc). Further exploration showed that the second principal component shows high loadings of descriptors related to descriptors with some relation to polarity or negativity, such as the *eta average electronegativity measure*, *mean atomic Sanderson electronegativity or topological surface area* (alvaDesc descriptors). The third principal component may be related to topological characteristics, e.g., there is a high factor loading of the *ring complexity index* and *distance* or *spanning indices* from *detour* or *Laplace matrix.*

#### 3.1.2. Explained Variance

Overall, 80% of the variance in the physicochemical descriptors could be explained by the first ten principal components, and 90% were reached when 25 components are included. The first principal component already accounted for around 36% of the total variance, and together with the second principal component, almost 50% of the variance could be explained. 

### 3.2. Perceptual Ratings of Odors

The dataset was comprised of 2207 participants [6]. A subset of 207 participants was excluded due to artefacts in the ratings (missing or unplausible values, e.g., values out of scale like “11” for detection threshold), which could not be clarified retrospectively. The resulting dataset therefore contains perceptual ratings for *n* = 2000 subjects (1166 males) aged 18 to 72 years (mean = 25.84, std = 6.08). All participants irrespective of general or specific reduction in olfactory performance were included in the analysis. As the participants had been recruited at the university campus, the sample was mostly comprised of university students. All subjects had been tested for detection threshold; a subset of participants additionally rated the perceived pleasantness and intensity (*n* = 1176) as well as the qualitative dimension of the odors (*n* = 976). The odors were presented in groups of 178 to 200 subjects (see Table 2 and Table 3). The mean rating of pleasantness ranged from the lowest pleasantness of mean = 2.25 for Isovaleric acid (std = 1.66) to the highest ratings for Lyral (mean = 5.59, std = 1.65) and Muscone (mean = 5.58, std = 1.69). The molecule with the highest rating of intensity (mean = 6.26, std = 1.69) was 1-Octen-3-one, followed closely by Trans-2-Nonenal (mean = 6.22, std = 1.70); the lowest ratings were given for Bacdanol (mean = 3.13, std = 1.76) and Sandranol (mean = 3.18, std = 1.61). The detection threshold was tested in groups of 276 to 376 participants. The mean detection threshold ranged from 4.19 for Geraniol (std = 1.70), which is equivalent to a dilution of 1:10^4^, to 7.29 for Isobutyraldehyde (dilution of 1:10^7^; std = 1.04).

#### 3.2.1. Pleasantness Ratings

**Figure 3:**
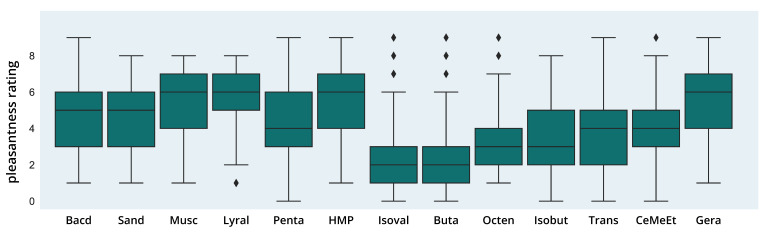
Pleasantness Ratings. The figure shows boxplots for pleasantness ratings (from 0 = extremely unpleasant to 9 = extremely pleasant) of different odor molecules. Rhombs display outliers.

A robust one-way Welch ANOVA showed significant differences in pleasantness ratings for the odor molecules in the dataset (F(12,983.25) = 80.69; *p* < 0.001, see Figure 3) with a high effect size (partial $\eta^{2}$ = 0.26). Games–Howell post hoc comparisons showed significant differences for 56 out of 78 possible comparisons with mostly high or very high effect sizes ranging from Cohen’s d = 0.45 (Bacdanol > Cedrylmethylether) to *d* = 2.02 (Lyral > Isovaleric acid). No significant differences were found within the group of odors with the highest ratings of pleasantness (median = 6): Lyral (5.59), Muscone (5.58), 3-Hydroxy-2-Methyl-4-pyrone (5.5) and Geraniol (5.49). Similarly, no differences were found between three of the four most unpleasant odors (median = (2; 3)): Isobutyraldehyde (3.57), 1-Octen-3-one (3.16) and 2,3-Butadione (2.75). Similarly, no significant differences were found for odors in the low to medium range (median = (4; 5)), and some odors neighboring the high or low pleasantness group did not differ significantly (see https://osf.io/k73ef/, accessed on 12 February 2021). 

#### 3.2.2. Intensity Ratings 

**Figure 4:**
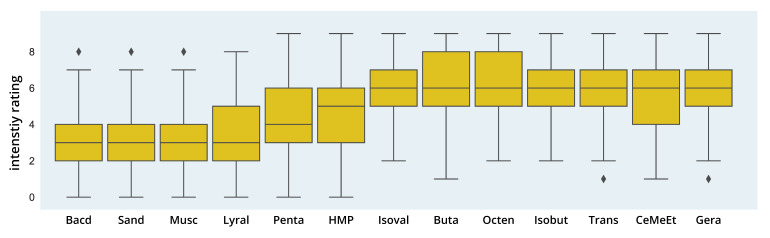
Intensity Ratings. The figure shows boxplots for intensity ratings (from 0 = not perceived to 9 = extremely intense) of different odor molecules. Rhombs display outliers.

A robust one-way Welch ANOVA showed significant differences in intensity ratings for the odor molecules in the dataset (F(12,983.40) = 106.14; *p* < 0.001, see Figure 4) with a high effect size (partial $\eta^{2}$ = 0.33). Games–Howell post hoc comparisons showed significant differences for 51 out of 78 possible comparisons with effect sizes ranging from d = 0.44 (1-Octen-3-one > Cedrylmethylether) to *d* = 1.86 (1-Octen-3-one > Sandranol). Similar to the pleasantness ratings, groups of high and low intensity odors without significant differences can be found: the highest intensity ratings (median = 6) were given for Cedrylmethylether (5.45), Geraniol (5.78), 2,3-Butadione (6.03), Isobutyraldehyde (6.04), Isovaleric acid (6.07), Trans-2-Nonenal (6.22) and 1-Octen-3-one (6.26). For the low intensity odors (median = 3), Bacdanol (3.13), Sandranol (3.18), Muscone (3.31) and Lyral (3.44) showed no significant difference in intensity rating. 

#### 3.2.3. Detection Threshold

**Figure 5:**
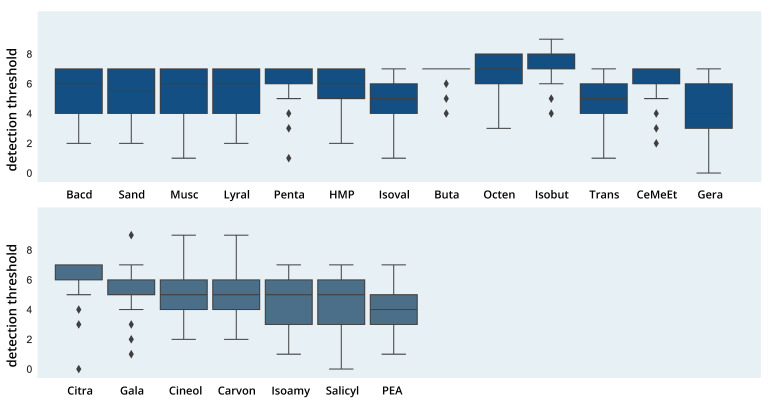
Detection Threshold. The figure shows boxplots for detection thresholds (dilutions from 0 = 1:10^0^ to 9 = 1:10^9^) of different odor molecules. Rhombs display outliers.

A robust one-way Welch ANOVA showed significant differences in detection threshold for the odor molecules in the dataset (F(20,2096.42) = 164.55; *p* < 0.001, see Figure 5) with a high effect size (partial $\eta^{2}$ = 0.27). Games–Howell post hoc comparisons showed significant differences for 139 out of 210 comparisons with effect sizes between d = 0.37 (Geraniol > Isoamylacetate) and d = 2.54 (Isobutyraldehyde > Phenyl ethyl alcohol). In analogy to pleasantness and intensity, some homogenous groups with similar detection thresholds can be found, e.g., Bacdanol (5.5), Sandranol (5.35), Muscone (5.33) and Lyral (5.44) again form a group with similar values, alongside 3-Hydroxy-2-Methyl-4-pyrone (5.88), Trans-2-Nonenal (5.32), Geraniol (5.08), Galaxolide (5.40), Isovaleric acid (5.02), 1,8-Cineol (5.20) and l-Carvon (5.17). For all comparisons, see https://osf.io/k73ef/, accessed on 12 February 2021. 

#### 3.2.4. Distribution of Pleasantness and Intensity Ratings

**Figure 6:**
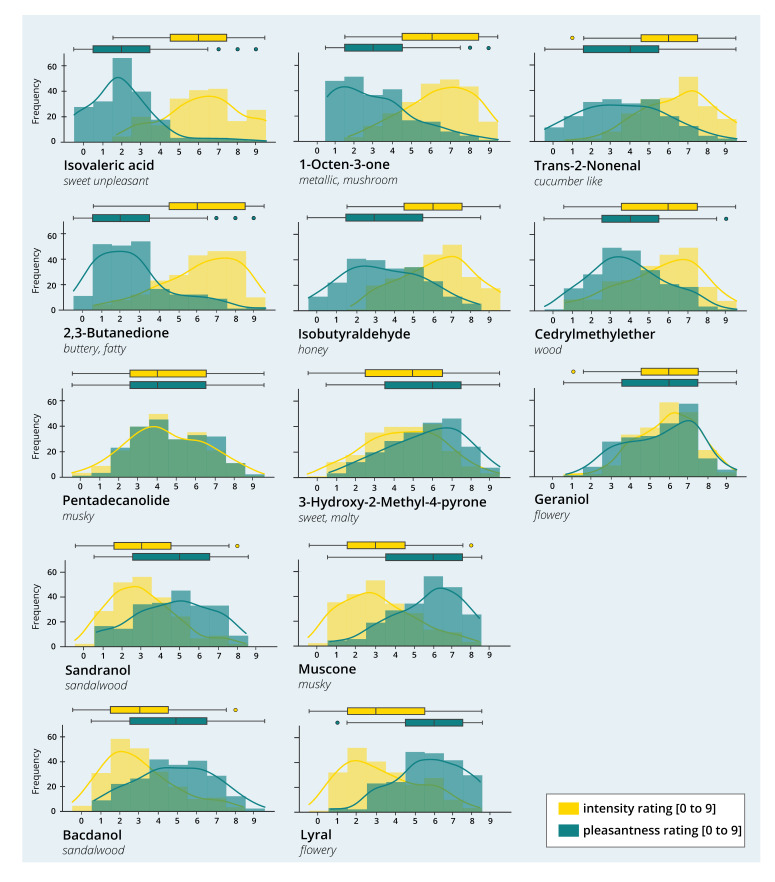
Distribution of Pleasantness and Intensity Ratings. The histogram plots illustrate the frequency (= total number of ratings) for perceptual ratings of pleasantness and intensity for each odor. The plots are arranged in subgroups according to low, medium and high values in pleasantness (ranging from 0 = extremely unpleasant to 9 = extremely pleasant) and intensity (ranging from 0 = not perceived to 9 = extremely intense). In order to give an idea of the qualitative impression of the odors, a semantic description is added for each. Circles indicate outliers.

Although large differences between perceptual ratings of pleasantness and intensity were found on the group level, a closer look at the distribution of the values reveals interindividual differences between participants’ ratings (see Figure 6 and Table 4 and Table 5). For example, one of the most pleasant odors, 3-Hydroxy-2-methyl-4-pyrone, shows a flat peak and broad distribution. While the median value of 6 is above the mean of the scale (4.5) for pleasantness, still 25% of the values fall in the range between 1 and 3, which corresponds to a quite unpleasant rating, and another 25% in the range of 4–6, i.e., neutral to moderately pleasant. A similarly broad distribution with an IQR spanning at least four values can also be seen for most of the low to medium pleasant odors: Trans-2-Nonenal, Isobutyraldehyde, Pentadecanolide, Sandranol and Bacdanol. For those odors, the values spread almost symmetrically around the median value 4 or 5, e.g., for Trans-2-Nonenal, the density curve of pleasantness ratings is very flat, with 50% of the values falling in the range of 0–4, and the other 50% in the range of 4–9. Interestingly, the intensity ratings for those odors generally show a narrower distribution and higher peaks, except for Pentadecanolide, which shows an almost identical (and equally broad) curve to the pleasantness ratings. In comparison, the steepest curves for pleasantness can be found for the two most unpleasant odors Isovaleric acid and 2,3-Butadione, and for the three most pleasant odors Geraniol, Lyral and Muscone. 

#### 3.2.5. Interaction of Pleasantness and Intensity Ratings

Noticeable in the visual inspection of the graphs is that there seems to be a negative association between pleasantness and intensity ratings (correlation coefficients are discussed in the next paragraph). For the six low to medium pleasant odors Isovaleric acid, 1-Octen-3-one, Trans-2-Nonenal, 2,3-Butadione, Isobutyraldehyde and Cedrylmethylether (first two rows in Figure 6), a low or medium pleasantness is accompanied by a high intensity rating. For Sandranol, Bacdanol, Muscone and Lyral, a medium to high pleasantness is complemented by a low intensity rating. As mentioned above, for Pentadecanolide, which has a medium or neutral pleasantness on average, the curves for pleasantness and intensity overlap almost completely. Somewhat inconsistently, two of the pleasant odors, 3-Hydroxy-2-Methyl-4-pyrone and Geraniol, show higher ratings for pleasantness, but also for intensity. 

#### 3.2.6. Correlations among Perceptual Ratings

**Figure 7:**
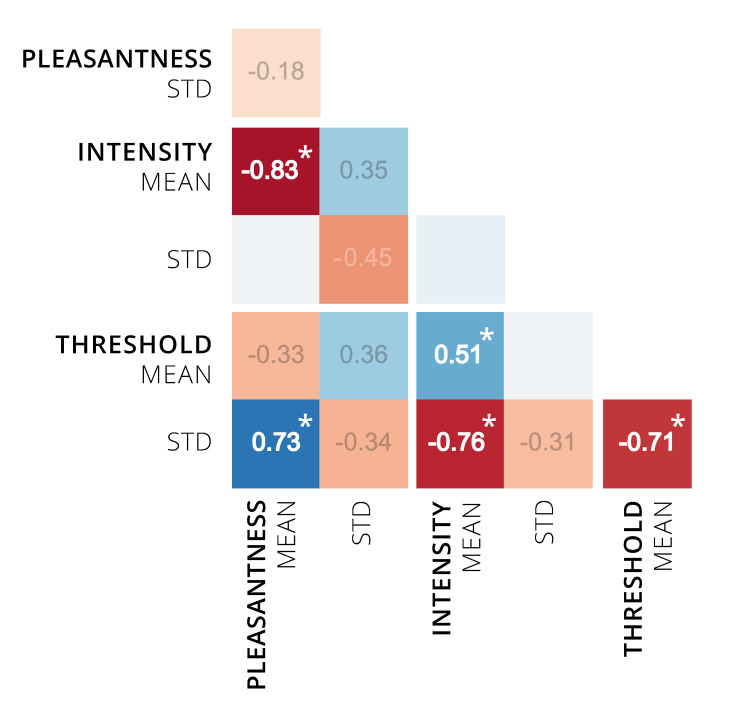
Correlation Matrix of Perceptual Ratings. Pearson correlation coefficients for associations between pleasantness (*n* = 13 odor molecules), intensity (*n* = 13) and detection threshold (*n* = 20). Highlighted in bold and with asterisk are significant correlations to the level of *p* < 0.05 (two-tailed *t*-test, uncorrected).

Pearson correlation coefficients show relations between the perceptual ratings of pleasantness, intensity and detection threshold (see Figure 7). The strong negative association (*r* = −0.83) that can be seen between the mean pleasantness and mean intensity ratings substantiates the observation from the distribution plots. Additionally, the mean intensity was positively related to the mean detection threshold (*r* = 0.51), i.e., the odors were rated as more intense if they were more easily detected (at a higher threshold). Interestingly, the standard deviation of the detection threshold also showed a positive correlation with the mean pleasantness (*r* = 0.73) and a negative correlation with the mean intensity (*r* = −0.76) and the mean detection threshold (*r* = −0.71). Or, put differently, if an odor is harder to detect on average, it also shows a broader distribution of detection threshold between individuals and the odor is rated as less intense on average. This association with intensity may result from a larger percentage of participants who have difficulties detecting the odor at all.

#### 3.2.7. Correlations of Perceptual Ratings with Vapor Pressure

Spearman correlation coefficients show a strong correlation between vapor pressure estimation and odor intensity (*r* = 0.82, *p* < 0.001) as well as a negative association with pleasantness (*r* = −0.68, *p* = 0.01). For detection threshold, no association with vapor pressure could be identified (*r* = 0.08, *p* = 0.80).

#### 3.2.8. Associations with Qualitative Ratings

**Figure 8:**
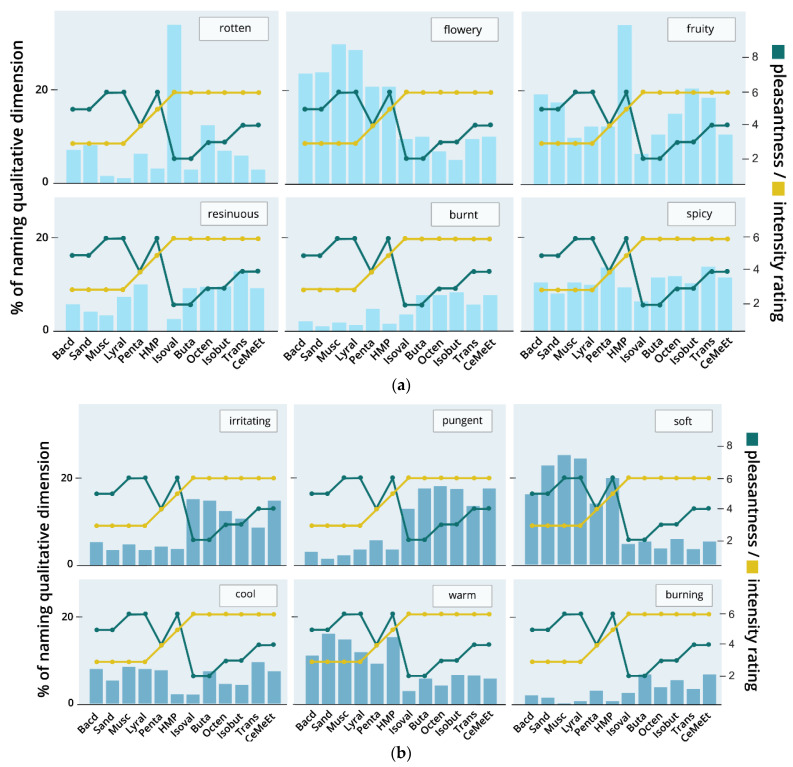
Qualitative Ratings. The figure shows the frequency (in %) of naming qualitative descriptors for each odor. The participants were asked to name the two descriptors that, in their opinion, fit best to each odor from a list of 12 qualitative descriptions (rotten, flowery, fruity, resinous, burnt, spicy, irritating, pungent, soft, cool, warm, burning; for details see Croy et al. [6]). Each subplot represents one of the qualitative descriptors, split into olfactory (**a**) and trigeminal (**b**) odor descriptions. To facilitate the discovery of associations between pleasantness, intensity and qualitative ratings, the median values for pleasantness and intensity are drawn as yellow and green lines in the plots.

In order to gain more insight on why some odors have more similar ratings of pleasantness and intensity than others, we investigated the qualitative ratings for the odors. The frequency of naming each of the 12 verbal descriptors (*rotten, flowery, fruity, resinous, burnt, spicy, irritating, pungent, soft, cool, warm, burning*) is visualized in Figure 8. The frequencies give an ambiguous picture. Some of the qualitative descriptors show a more or less equally distributed frequency of mention for all odor molecules, for example, “cool” and “spicy”, and therefore do not seem to discriminate easily between the odors. For other descriptors, there appears to be some trend of association with pleasantness and intensity. In the olfactory descriptors, those odors that were more frequently named as “flowery” were also rated as more pleasant. Interestingly, Isovaleric acid was named as “rotten” by more than 20% of the subjects, and this is also reflected in a low pleasantness rating and a high intensity rating. Vice versa, the highest pleasantness ratings were accompanied by a low percentage of naming the odor as “rotten”. Another striking peak is shown for “fruity” for the odor 3-Hydroxy-2-methyl-4-pyrone. In the trigeminal domain, those descriptors that are unpleasant show an association with a high intensity rating: both “irritating” and “pungent” were named more frequently for those odors with a high intensity, and the two descriptors “soft” and “warm” were named more often for odors with a low intensity. “Soft” and “warm” also show some relation to a higher pleasantness rating. The results indicate that the olfactory descriptors are better represented in the results for pleasantness, whereas intensity is more prominent for the trigeminal descriptors.

### 3.3. Associations between Perceptual Ratings and Physicochemical Odor Space

**Figure 9:**
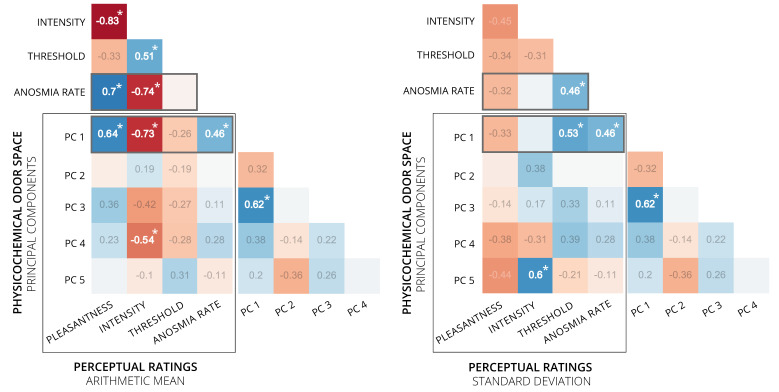
Correlation Matrices. Pearson correlation coefficients for associations between principal components (PC1 to PC5) from physicochemical odor space and arithmetic mean (**a**) and standard deviation (**b**) of perceptual ratings for pleasantness (*n* = 13 odor molecules), intensity (*n* = 13) and detection threshold (*n* = 20). Highlighted in bold and with asterisk are significant correlations to the level of *p* < 0.05 (two-tailed *t*-test, uncorrected).

**Figure 10:**
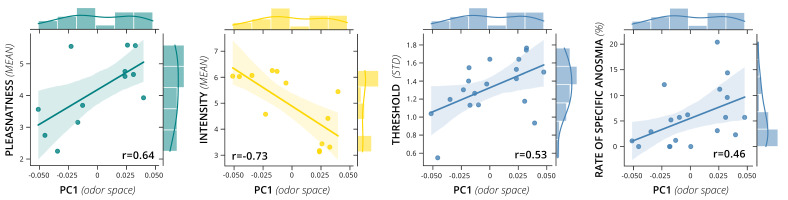
Associations between Physicochemical Odor Space and Perceptual Ratings. The figure shows correlations between the mean values for (**a**) pleasantness (*n* = 13 odor molecules) and (**b**) intensity ratings (*n* = 13), (**c**) standard deviation of detection threshold (*n* = 20) as well as (**d**) the rate (%) of specific anosmia (*n* = 20) with the first principal component obtained from the odor space.

As a last step in our analysis, for the 20 odor molecules investigated we calculated Pearson correlation coefficients for the association between the values of the first five principal components from the odor space and the respective values of the perceptual ratings of pleasantness, intensity, detection threshold and the rate of specific anosmia as obtained from Croy et al. [6] (see Figure 9 and Figure 10). For the first principal component (PC) of the odor space, a positive association was found for the mean pleasantness (*r* = 0.64) and a negative association with mean intensity (*r* = −0.73). Therefore, the variation in PC1 accounts for some variation in those variables—although the results must be taken with care due to the very small number of odor molecules in the correlation calculation (13 odors for pleasantness and intensity ratings). The mean detection threshold showed no correlation with PC1. However, a positive association was found with the standard deviation of detection threshold (*r* = 0.53) and with the rate of specific anosmia (*r* = 0.46). Interestingly, the rate of specific anosmia shows the same correlation “pattern” as PC1 with the means and standard deviations of the perceptual ratings (compare third and fourth row from top in both correlation matrices). For example, the rate of specific anosmia correlates positively (r = 0.7) with mean pleasantness and negatively with mean intensity ratings (*r* = −0.73). Further associations were found between PC4 and the mean intensity (*r* = −0.54) and between PC5 and the standard deviation of intensity (*r* = 0.60). Additionally, PC1 and PC3 showed a correlation of *r* = 0.62. 

## 4. Discussion

### 4.1. Discussion of Results

Following the example of previous authors [2,3,8,16], we examined more than 20,000 molecule properties to build our own physicochemical odor space. In our case, more than 80% of the variance could be explained by the first ten principal components, and almost 50% by the first two PCs alone. These values are very similar to previous approaches with usually smaller descriptor sets, and therefore support the idea that there is high redundancy between the descriptors and a much smaller set of descriptors is sufficient to characterize olfactory stimuli. Additionally, consistent with previous studies [2,8], the first principal component was associated with descriptors indicative of molecular weight, size, or complexity. For the second principal component, different interpretations for the “label” of the dimension have been found: Mainland [2] characterized the second dimension as the “linearity” of odor molecules, i.e., the chain-length of the molecule. In our odor space, the highest loadings were found for descriptors that are related to negativity or polarity, indicating a dimension that differentiates the “chemical behavior” more than topological characteristics. Some relation to length or ring complexity was found for the third principal component in our odor space. 

Although these findings help to explore the dimensions of olfaction, they can only take us so far. The more descriptors are included in the calculation, the more difficult it becomes to interpret the contribution of each descriptor to the different dimensions. In our odor space, the 200 descriptors with the highest loadings for PC1 all showed very similar values (less than +/− 2.5% from mean). This makes any interpretation of content challenging, and our conclusions about tentative labels for the dimensions may have to be revised if all high factor loadings are taken into account. New hypotheses and further analyses are needed to find the common content of these descriptors. Still, the odor space is a helpful tool to relate the dimension of physicochemical properties with the perceptual dimensions of olfaction. 

With regard to the perceptual ratings, our dataset of 2000 naïve subjects showed significant differences in perceived pleasantness and intensity on the group level with a high association between both perceptual dimensions. Odors with a low intensity showed higher pleasantness ratings, while a high intensity was perceived for unpleasant odors. Although moderate to high effect sizes could be seen for the differences in pleasantness and intensity ratings between the odors, these need to be treated more conservatively as each group of participants rated several (but not all) odors. Therefore, similarities in rating patterns may have occurred within the groups and between-group differences may be over-interpreted. For example, the musky odor Muscone and the flowery odor Lyral were presented to the same participants and showed similar ratings in pleasantness, intensity and detection threshold. Therefore, cross-influences cannot be ruled out, although efforts had been made in the original study to rule out influences between the successive ratings, e.g., the odors were presented precisely for the ratings and a short time only to avoid habituation. The same may hold true for the two sandalwood odors Sandranol and Bacdanol, although similar ratings also seem plausible as both are sandalwood odors. 

The data for this study were originally collected in the context of investigating the prevalence of specific anosmia and its role as a peripheral adaptive filtering mechanism [6]. There, it has already been shown that odors with a higher molecular weight showed higher rates of specific anosmia. In this study, we found that the extent of interindividual difference (standard deviation) in detection threshold is related to the first principal component of the odor space. This shows that the first PC of the physicochemical odor space is not indicative of the absolute value of the concentration threshold for detection, but of the percentage of participants who deviated from this value, i.e., among others, those who were anosmic for this specific odor. Interestingly, there were striking differences for the rates of specific anosmia for the two sandalwood odors, Bacdanol (20.4%) and Sandranol (3.1%), which have the same CAS registry number and are sometimes treated as synonyms in databases such as PubChem. In practice, substances with the same CAS number can have different distributions of isomers that can lead to different and distinguishable olfactory percepts [37]. In our example, Sandranol shows a different distribution of enantiomers (i.e., a form of isomer that is an exact mirror of the same chemical compound, but cannot be brought into congruence) in the odor solution depending on the synthesis method and odor concentration (*private communication*). This makes Sandranol the “stronger” odor compared to Bacdanol. However, since our calculation of the physicochemical descriptors does not distinguish between isomers, we cannot account for the perception difference in the two odors. In summary, the typically unknown composition of odors in a solution provides another source of uncertainty which contributes to variance in the perceptual ratings.

Further insight on why certain odors may have been rated similarly or differently was found in the qualitative descriptions. Explorative analyses showed relations between odor quality and the perceived pleasantness, e.g., positive descriptions such as “flowery” were given more often for those odors that were rated as pleasant and negative descriptions (e.g., “rotten”) were more likely associated with unpleasant odors. Similarly, intensity ratings were also influenced by the trigeminal nature of an odor. Those odors that were perceived as “pungent” or “irritating” were also rated as more intense than other odors. However, each qualitative descriptor was named for each odor, sometimes rather evenly distributed, and the overall picture remains unclear. This may partly be attributed to the study design, as each participant had to choose two out of twelve qualitative descriptors that fit best, but not necessarily ruling out that more than those two descriptors fit the odor. This way, it is possible that an odor that is perceived as irritating and pungent could not (additionally) be rated as flowery if this seemed the less relevant dimension. 

While we were able to find significant differences on the group level, there were also striking interindividual variations in the perceptual ratings of the different odors. Although certain odors tend to be rated as pleasant or unpleasant more often, e.g., the flowery odors Lyral and Geraniol, even for those odors there were notable percentages of participants who did not like the smell, and vice versa, for the most unpleasant odor there were still pleasant ratings. This does not come as a surprise, considering that especially pleasantness can be seen as a somewhat ambiguous dimension. Olfactory perception is influenced by (among other things) the familiarity of the odor [3], expectations about the odor source [38], interoceptive sensations [39], perceptual or verbal abilities [27] and personality traits of the individual [25,40]. Olfactory perception can even be tracked down as far as to prenatal influences and development (for the interested reader, see [41,42]) and is closely linked to gustation [42]. This “noise” in the data must be taken into account when it comes to the association with the physicochemical odor space. For example, concerning the familiarity of odors and verbal abilities, the educational level of study participants may also influence olfactory ratings. For the perceptual ratings analyzed here, only multiple choice questions in an easy language had to be answered, which most likely did not enhance variance in the sample mostly comprised of university students. Still, this influence should be taken into account, especially in studies where free verbal descriptions have to be given by the participants.

A perfectly accurate prediction of an olfactory percept from the molecular structure is, thus, unlikely to be found, especially on the individual level. Still, our results support previous findings on the relation between odor pleasantness and intensity with molecular properties on the group level [3,8,10,16]. Additionally, we showed that the variation in detection threshold is related to the first principal component of the odor space, supporting the previous finding that the rate of specific anosmia is related to molecular weight [6]. Moreover, the rate of specific anosmia was found to be similar to PC1 of the odor space, having a positive relation to pleasantness and a negative association with intensity. With respect to the vapor pressure, we have found a strong positive association with the perceived intensity, but not with detection threshold. This is plausible because it may be hypothesized that higher vapor pressure leads to higher concentrations of molecules in the olfactory cleft and therefore to a larger likelihood of the binding of molecules to olfactory receptors and, as a consequence, increased intensity perception. Detection threshold, on the other hand, may be more dependent on the individual expression of certain olfactory receptors.

### 4.2. Limitations

Some limitations shall be discussed. As a first important aspect, the exploratory nature of this study has to be pointed out. The main goal of this study was to focus on the olfactory perception of non-expert raters and test the validity of structure–percept associations in this sample. As previous literature on naïve samples is rare, the investigation of distributions, as well as differences and interactions in the perception of pleasantness, intensity, detection threshold and qualitative ratings, was carried out in an exploratory and hypothesis generating manner. The results discussed here will need confirmation in another study and with a larger set of chemically different odor molecules. 

The number of odor molecules must be seen as a second limitation of our study. This caveat results from the fact that our dataset was originally collected and investigated for a different purpose (i.e., studying the prevalence and influencing factors of specific anosmia; see [6]) Although our results point to very similar directions as previous associations between pleasantness, intensity and, for example, molecular weight, it has to be stressed that with our sample of 13 to 20 molecules the study does not have sufficient statistical power to draw reliable conclusions and results need to be taken with caution. While the general association between odor space and corresponding perception seems plausible and has been found before, the high correlations found here must be questioned, especially considering the large interindividual variations in perceptual ratings found in our data.

Additionally, the odors were presented in groups of participants that received the same two or three odors. This may lead to an over interpretation of significant differences—or missing differences—between perceptual ratings for those odors that were presented together in one group. Furthermore, inhomogeneities of variance may have occurred from the different numbers of participants that rated each odor. Although these deviations were small and have been accounted for by using robust statistical methods such as Welch ANOVA, it has to be named as a limitation.

To summarize, we believe that our results are an important indication on the extent to which previous findings for olfaction experts also apply to naïve raters. The findings must, however, be replicated in a larger collection of odors in order to be able to make definite statements. Open science practices can make an important contribution here in making psychophysical data available so that future research can build on existing datasets.

## 5. Conclusions

The physicochemical odor space developed from molecular descriptors is increasingly becoming a tool for studying the *stimulus-percept problem*. Although there is no standard procedure yet, for many investigations it has proven to be a helpful and reliable instrument to narrow down the vast and high-dimensional nature of olfactory stimuli. In addition, the odor space may also serve for choosing chemically diverse molecules for empirical investigations that aim to relate structure to perception. Our study built heavily on previous approaches and validates the method with a different and larger set of molecular descriptors. The resulting odor space as well as the psychophysical data will be made available on public platforms to push the further investigation of the central dimensions of olfaction.

Regarding the relationship between odor space and perceptual ratings, we showed that associations between odor structure and the corresponding perceptual ratings of pleasantness and intensity, as well as their perceptibility, i.e., detection threshold, can also be found in a sample of non-expert raters. In this respect, our dataset contributes to the enlargement of the pool of psychophysical data on olfactory perception, encompassing a large population of non-expert participants. While our study stands out with its large naïve sample and the results go in line with previous results, it is limited by the small set of odor molecules for which we obtained perceptual ratings. Future studies should look at a larger number of odor molecules to make profound conclusions about relationships between structure and perception. Furthermore, more information about influencing factors on the part of the study participants should be taken into account, as implied by the large interindividual variations found for the perceived pleasantness and intensity of odors. 

While advances in computational methods have made it possible to make increasingly accurate predictions from physicochemical structure to percept, it is noted that human olfactory perception is no analytical process of molecule detection, but is part of a multisensory integration of visual, auditory, haptic and social information from our environment. Moreover, the interpretation of sensory inputs is heavily influenced by top-down processes that are steered by memory, experience, interoception and interpersonal characteristics. The prediction of perception from odor stimulus structure can therefore only work to a certain degree, and perhaps physicochemical dimensions of individual molecules can be seen as a necessary but not sufficient condition to determine the corresponding percept of an individual. 

To put it in a nutshell, broad knowledge is currently created by the ongoing research on both the more *sensoric* part of olfaction, i.e., binding patterns on the receptor side, as well as the *perceptual* interpretation of olfactory stimuli in the light of situation and experience. Both paths are facilitated by the emergence of more and more sophisticated computational methods. Integrating the findings on these different levels of examination seems a promising path to further demystifying the complex nature of human olfactory perception and may have an impact on the development of electronic olfaction devices.

## Figures and Tables

**Table 1:** Overview of Odors. Listed are CAS registry number, trivial name, abbreviation used in plots and graphs and vapor pressure estimations (in mmHg at 25 °C; retrieved from PubChem * and ChemSpider **).

	CAS Number	Trivial Name	Abbreviation	Vapor Pressure
1	204-262-9	Salicylic ester	Salicyl	0.000078 *
2	99-49-0	l-Carvon	Carvon	0.115 *
3	431-03-8	2,3-Butadione	Buta	56.82 *
4	31906-04-4	Lyral	Lyral	0.0000857 **
5	5146-66-7	Citralva	Citra	0.0362 **
6	106-24-1	Geraniol	Gera	0.03 *
7	78-84-2	Isobutyraldehyde	Isobut	170 *
8	503-74-2	Isovaleric acid	Isoval	0.44 *
9	628-46-6	Isoamylacetate	Isoamy	0.058 *
10	118-71-8	3-Hydroxy-2-methyl-4-pyrone	HMP	0.000326 *
11	470-82-6	1,8-Cineol	Cineol	1.90 *
12	956-82-1	Muscone	Musc	0.000469 **
13	6602-64-8	Galaxolide	Gala	0.000889 **
14	28219-61-6 ^1^	Sandranol	Sand	0.0000718 **
15	28219-61-6 ^1^	Bacdanol	Bacd	0.0000718 **
16	18829-56-6	Trans-2-nonenal	Trans	0.317 **
17	19870-74-7	Cedrylmethylether	CeMeEt	0.0201 **
18	106-02-5	Pentadecanolide	Penta	0.0000517 **
19	60-12-8	Phenylethylalcohol	PEA	0.09 *
20	3391-86-4	1-Octen-3-one	Octen	0.238 **

^1^ Note: Sandranol and Bacdanol are listed with the same CAS registry number and therefore correspond with the same structural descriptors in further analyses.

**Table 2:** Overview of Subject Groupings, Odorants and Perceptual Ratings obtained.

Subjects	Odorants Used for Testing							Perceptual Ratings			
								*thr*	*int*	*pl*	*qual*
1–200	Isoval	Trans	-	-	-	-	-	X	X	X	X
201–400	HMP	Penta	CeMeEt	-	-	-	-	X	X	X	X
401–600	Sand	Bacd	Buta	-	-	-	-	X	X	X	X
601–800	Lyral	Musc	-	-	-	-	-	X	X	X	X
801–1000	Gera	-	-	-	-	-	-	X	X	X	
1001–1200	PEA	Gera	Cineol	-	-	-	-	X			
1201–1400	Carvon	Isoamy	Salicyl	-	-	-	-	X			
1401–1600	Citra	Gala	-	-	-	-	-	X			
1601–1800	Isobut	Octen	-	-	-	-	-	X	X	X	X
1801–2000	PEA	Citra	Cineol	Isoamy	Salicyl	Gala	Carvon	X			

Abbreviations: thr = threshold, int = intensity, pl = pleasantness, qual = quality.

**Table 3:** Descriptive Statistics for Detection Threshold and Pleasantness and Intensity Ratings.

	Pleasantness				Intensity			Detection Threshold			
	*N*	*mean*	*median*	*std*	*mean*	*median*	*std*	*N*	*mean*	*median*	*std*
Bacd	200	4.76	5	1.88	3.13	3	1.76	300	5.50	6	1.53
Sand	200	4.61	5	1.88	3.18	3	1.61	300	5.35	6	1.43
Musc	200	5.58	6	1.69	3.31	3	1.79	300	5.33	6	1.77
Lyral	200	5.59	6	1.65	3.44	3	1.86	300	5.44	6	1.64
Penta	200	4.67	4	1.87	4.42	4	1.89	300	6.19	7	1.18
HMP	200	5.55	6	1.88	4.58	5	1.88	300	5.88	6	1.30
Isoval	178	2.25	2	1.66	6.07	6	1.86	300	5.02	5	1.20
Buta	200	2.75	2	1.87	6.03	6	1.89	300	6.78	7	0.55
Octen	200	3.16	3	1.85	6.26	6	1.69	300	6.82	7	1.13
Isobut	200	3.57	3	1.97	6.04	6	1.72	300	7.29	7	1.04
Trans	178	3.69	4	2.07	6.22	6	1.70	300	5.32	5	1.26
CeMeEt	200	3.93	4	1.79	5.45	6	1.94	300	6.28	7	0.93
Gera	198	5.49	6	1.75	5.78	6	1.51	376	4.19	4	1.70
Cineol	-	-	-	-	-	-	-	300	5.20	5	1.64
PEA	-	-	-	-	-	-	-	300	3.94	4	1.55
Carvon	-	-	-	-	-	-	-	300	5.17	5	1.37
Isoamy	-	-	-	-	-	-	-	300	4.76	5	1.40
Salicyl	-	-	-	-	-	-	-	300	4.77	5	1.74
Citra	-	-	-	-	-	-	-	300	6.22	7	1.14
Gala	-	-	-	-	-	-	-	300	5.40	6	1.50

**Table 4:** Interquartile Ranges for Pleasantness Ratings.

	*Bacd*	*Sand*	*Musc*	**Lyral**	*Penta*	*HMP*	*Isoval*	*Buta*	*Octen*	*Isobut*	*Trans*	*CeMeEt*	*Gera*
**+/− 1.5 IQR**	[1, 9]	[1, 8]	[1, 8]	[2, 8]	[0, 9]	[1, 9]	[0, 6]	[0, 6]	[1, 7]	[0, 8]	[0, 9]	[0, 8]	[1, 9]
**IQR**	3–6	3–6	4–7	5–7	3–6	4–7	1–3	1–3	2–4	2–5	2–5	3–5	4–7
**median**	5	5	6	6	4	6	2	2	3	3	4	4	6

**Table 5:** Interquartile Ranges for Intensity Ratings.

	*Bacd*	*Sand*	*Musc*	*Lyral*	*Penta*	*HMP*	*Isoval*	*Buta*	*Octen*	*Isobut*	*Trans*	*CeMeEt*	*Gera*
**+/− 1.5 IQR**	[0, 7]	[0, 7]	[0, 7]	[0, 8]	[0, 9]	[0, 9]	[2, 9]	[1, 9]	[2, 9]	[2, 9]	[2, 9]	[1, 9]	[2, 9]
**IQR**	2–4	2–4	2–4	2–5	3–6	3–6	5–7	5–8	5–8	5–7	5–7	4–7	5–7
**median**	3	3	3	3	4	5	6	6	6	6	6	6	6

**Figure A1:**
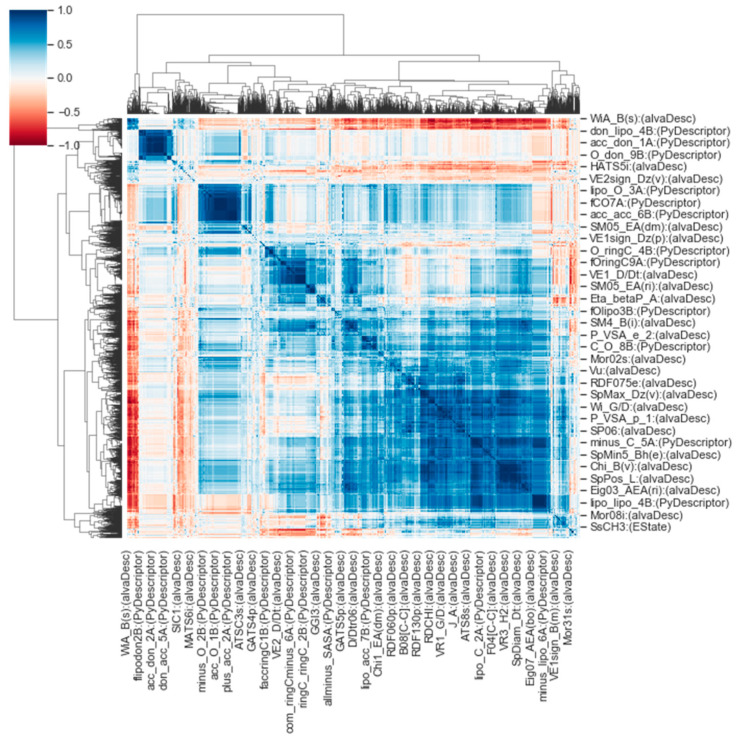
Clustered correlation matrix of all 4094 molecular descriptors. From visual inspection of the correlation matrix, it can be seen that there are large correlations between the descriptors, forming clusters of different sizes. To eliminate redundancies in the large descriptor collection, dimension reduction was performed using principal component analysis.

## Data Availability

The data presented in this study are openly available in Open Science Framework (OSF) at DOI: 10.17605/OSF.IO/E67DN at https://osf.io/e67dn/ (accessed on 12 February 2021).

## Appendix A

**Descriptor calculation**. For the development of a physicochemical property space, we used a list of 1565 odorants typically used in experiments and industry as provided by Khan et al. [8], listed by name and CAS registry number (CAS = Chemical Abstracts Service). Additionally, we added six odors used in the dataset of perceptual ratings [6] that were not included in the list from Khan et al., resulting in 1571 odors in total. As a first step, we retrieved the Simplified Molecular Input Line Entry Specification (SMILES) for each odorant using the *webchem* package in RStudio© (version 1.2.5033, R version 3.6.2). *webchem* retrieves molecular properties from online chemical databases such as PubChem and ChemSpider via their CAS number, IUPAC or trivial name, InChiKey or other ids such as PubChem’s individual identification number [31]. For 1389 odorants, *webchem* was able to identify SMILES from CAS number or name (see https://osf.io/zv3te/, accessed on 12 February 2021). For the remaining 182 odors (mostly fragrance oils), no molecule could be clearly identified by the package. Therefore, we removed those odors from our dataset. We then used the *Online chemical database* (OCHEM, https://ochem.eu/, accessed on 12 February 2021), which allows the calculation of a large number of descriptors for the previously obtained SMILES [32]. We chose to calculate the 16,251 *PyDescriptor* descriptors [43], the 5305 *alvaDesc* descriptors [44] and a prediction of melting point and water solubility provided by OCHEM (see https://osf.io/vyr97/, accessed on 12 February 2021). In total, we calculated 21,609 descriptors.

**Preprocessing**. For the resulting 21,609 molecular descriptors, we identified and removed all descriptors containing infinite or missing values for any molecule and descriptors with zero values for more than 80% of the 1389 molecules. Furthermore, we dropped all descriptors without noteworthy correlations (no correlation r > 0.3 with any other descriptor). This resulted in the final physicochemical odor space with 4094 descriptors for each of the 1389 molecules.
